# A Population of CD4^+^CD8^+^ Double-Positive T Cells Associated with Risk of Plasma Leakage in Dengue Viral Infection

**DOI:** 10.3390/v14010090

**Published:** 2022-01-05

**Authors:** Esther Dawen Yu, Hao Wang, Ricardo da Silva Antunes, Yuan Tian, Rashmi Tippalagama, Shakila U. Alahakoon, Gayani Premawansa, Ananda Wijewickrama, Sunil Premawansa, Aruna Dharshan De Silva, April Frazier, Alba Grifoni, Alessandro Sette, Daniela Weiskopf

**Affiliations:** 1 Center for Infectious Disease and Vaccine Research, La Jolla Institute for Immunology, La Jolla, CA 92037, USA; dyu@lji.org (E.D.Y.); rantunes@lji.org (R.d.S.A.); ytian@fredhutch.org (Y.T.); rtippalagama@lji.org (R.T.); dslv90@yahoo.com (A.D.D.S.); afrazier@lji.org (A.F.); agrifoni@lji.org (A.G.); 2 School of Medicine, University of California San Diego, La Jolla, CA 92037, USA; haw040@health.ucsd.edu; 3 Genetech Research Institute, Colombo 00800, Sri Lanka; shaku.alahakoon@gmail.com; 4 Colombo North Teaching Hospital, Ragama 11010, Sri Lanka; gavisprema@gmail.com; 5 National Institute of Infectious Diseases, Angoda 10620, Sri Lanka; anandawijewickrama@hotmail.com; 6 Department of Zoology and Environment Sciences, University of Colombo, Colombo 00700, Sri Lanka; suviprema@gmail.com; 7 Department of Paraclinical Sciences, Faculty of Medicine, General Sir John Kotelawala Defence University, Mount Lavinia 10390, Sri Lanka; 8 Department of Medicine, Division of Infectious Diseases and Global Public Health, University of California, La Jolla, CA 92037, USA

**Keywords:** CD4^+^, CD8^+^, double positive, T cells, infectious diseases, dengue, plasma leakage, transcriptomic analysis

## Abstract

According to the WHO 2009 classification, dengue with warning signs is at the risk of developing severe form of dengue disease. One of the most important warning signs is plasma leakage, which can be a serious complication associated with higher morbidity and mortality. We report that the frequency of CD4^+^CD8^+^ double-positive (DP) T cells is significantly increased in patients at risk of developing plasma leakage. Transcriptomic analysis demonstrated that CD4^+^CD8^+^ DP cells were distinct from CD4^+^ Single Positive (SP) T cells but co-clustered with CD8^+^ SP cells, indicating a largely similar transcriptional profile. Twenty significant differentially expressed (DE) genes were identified between CD4^+^CD8^+^ DP and CD8^+^ SP cells. These genes encode OX40 and CCR4 proteins as well as other molecules associated with cell signaling on the cell surface (*NT5E*, *MXRA8*, and *PTPRK*). While comparing the profile of gene expression in CD4^+^CD8^+^ DP cells from patients with and without warning signs of plasma leakage, similar expression profile was observed, implying a role of CD4^+^CD8^+^ DP cells in plasma leakage through a quantitative increase rather than functional alteration. This study provided novel insight into the host immune response during the acute febrile phase of DENV infection and the role of CD4^+^CD8^+^ DP T cells in the pathogenesis of plasma leakage.

## 1. Introduction

Dengue fever (DF) is one of the major re-emerging infectious diseases and the most prevalent arthropod-borne viral disease in humans [1]. The Center for Diseases Control and Prevention (CDC) estimates that around 400 million people get infected annually, and approximately 3 billion of the world’s population live in dengue-endemic areas [2]. The 1997 WHO classification [3] distinguished four main categories of dengue disease: non-classical DF, classical DF, dengue hemorrhagic fever (DHF), and dengue shock syndrome (DSS). The diagnosis of DHF required the presence of four criteria: fever, thrombocytopenia (<100,000 platelets/mm^3^), as well as both hemorrhagic and plasma leakage manifestations. Several studies reported that this classification did not fully correlate with disease severity [3,4,5] and had limited sensitivity in detecting severe dengue cases that require advanced medical care [6,7]. As a consequence, a new WHO classification scheme was released [1] in 2009 that divides dengue into two categories: non-severe and severe dengue, with non-severe dengue further subcategorized as dengue without warning signs and dengue with warning signs. Plasma leakage is one of the most important warning signs, and it can be defined as abnormal extravascular accumulation of body fluids diagnosed clinically or radiologically and/or hemoconcentration (rise in hematocrit (HCT) ≥ 20% of the patient’s baseline level or a drop in HCT ≥ 20% of the baseline level following rehydration) [1].

The most dangerous complication of dengue infection is the dengue shock syndrome, a consequence of severe intravascular volume depletion from plasma leakage due to increased vascular permeability and decreased intravascular osmolarity. The severity of plasma leakage varies among patients, and failure to promptly identify and treat this warning sign of dengue is related to high mortality [8]. Thus, it is important to identify risk factors for the shock syndrome and to predict progression of dengue to more severe disease, such as plasma leakage or shock during the acute febrile phase.

Studies reported that female, infant, elderly patients, and those with comorbidities are prone to have more severe infection/shock syndrome [9,10]. Virus serotype, secondary infections, and laboratory tests, such as platelet count, serum albumin, aspartate aminotransferase (AST), and alanine aminotransferase (ALT) levels, may be also related and should be monitored during the febrile phase of illness [10]. However, these risk factors are not sensitive enough to be used in a clinical setting to predict severe disease, and more factors are yet to be determined. Thus, while several studies reported symptomatologic associations, the molecular mechanisms involved in plasma leakage progression to shock are still not well defined.

Here, we examined whether we could define changes in T-cell subset composition as a potential marker of plasma leakage progression to shock and also as a way to probe the associated underlying molecular mechanisms. We report that a population of CD4^+^CD8^+^ double-positive (DP) T cells detectable in the peripheral blood is associated with risk of plasma leakage in dengue disease. We further investigated the specific gene expression profiles associated with this subpopulation compared to other T cell subsets and as a function of disease severity.

## 2. Materials and Methods

### 2.1. Study Cohort and PBMC Isolation

The aim of this study was to investigate the association of the gene expression profiles and phenotypic attributes of CD4^+^CD8^+^ double-positive (DP) cells with plasma leakage in dengue diseases by RNA-sequencing and flow cytometry. The blood samples from patients with acute dengue virus (DENV) infection were collected upon diagnosis or admission with a median of 4 days after onset of fever (interquartile range (IQR): 4.0–6.0 days), in the North Colombo Teaching Hospital, Ragama in Gampaha District, Sri Lanka, and the National Institute of Infectious Diseases, Gothatuwa, Angoda, Sri Lanka, between 2010 and 2016. This study was approved by the appropriate local Ethics committees (see below for details). Each participant provided informed consent and was assigned a study identification number with clinical information recorded. The diagnosis of dengue infection was confirmed if at least one of the following criteria was met in acute phase serum: (1) positive reverse transcription polymerase chain reaction (RT-PCR) of DENV RNA, (2) positive serology for dengue IgM, or (3) positive dengue-specific non-structural antigen-1 (NS1). All patients were screened to ensure no history of anemia, HIV/HBV/HCV infection, or significant systemic disease. One group comprises patients that were diagnosed as dengue without warning sign of plasma leakage (D-L) cases, while the other group was classified as dengue with warning sign of plasma leakage (D+L) cases upon discharge. Plasma leakage in this study was defined as abnormal extravascular accumulation of body fluids and diagnosed according to 2009 WHO classification of dengue [1]. In the current study, no signs of plasma leakage were observed at initial diagnosis, and all the plasma leakage cases were confirmed by ultrasound several days after diagnosis/admission. The details of the patients recruited in this study were summarized in (Table 1). PBMCs were isolated at the Genetech Research Institute from whole blood as previously described [11] and cryopreserved for further analysis.

There was no difference in demographic features between the two groups. The median age was 26.0 (IQR (interquartile range): 19.0–43.0) years in the D-L group and 29.0 (IQR: 23.7–35.5) years in the D+L group. Females accounted for 30.3 % in D-L group and 42.6% in the D+L group. All blood samples were taken and sent for blood tests (shown in Table 1) during the acute febrile phase of DENV infection with a median of 5.0 (IQR: 4.0–6.0) days from fever onset in the D-L group and a median of 4.0 (IQR: 4.0–6.0) days from fever onset in the D+L group.

There were no significant differences in the results of dengue serology or PCR tests between the two groups: 57.6% of patients in the D-L group and 66.7% in the D+L group were IgM positive, 72.7% in the D-L group and 76.4% in the D+L group were IgG positive, and 30.3% in the D-L group and 46.9% in the D-L group were PCR positive. A total of 72.7% of patients in the D-L group and 76.4% in the D+L group had at least one previous episode of dengue infection.

Laboratory results showed a higher severity of disease complications in the D+L group in the acute febrile phase, including lower platelet counts (D+L: 39.0 (IQR: 13.0–90.0) ×1000/mm^3^ vs. D-L: 74.0 (IQR: 36.0–120.0) ×1000/mm^3^, *p* = 0.007) and worse transaminitis (AST: 230.0 (IQR: 96.0–309.7) U/L in D+L group vs. 113.0 (IQR: 43.5–216.7) U/L in D-L group, *p* = 0.017; ALT: 150.0 (IQR: 62.0–280.4) U/L in D+L group vs. 90.5 (IQR: 32.2–168.6) U/L in D-L group, *p* = 0.028). No difference in hematocrit, a marker of hemoconcentration, was seen in the acute febrile phase of DENV infection (42.3% (IQR: 39.5–46.1%) in the D+L group vs. 40.9% (37.2–44.5%) in the D-L group, *p* = 0.139).

### 2.2. Dengue Serology and PCR Tests

DENV serology tests were performed with anti-DENV IgG and IgM ELISA as previously described [12]. Reverse transcription-PCR was performed by using the DV1 and DV3 primer set [13] and the ALD 1 and ALD 2 primer set [14] in one reaction with details specified previously [15].

### 2.3. Flow Cytometry Analysis and Cell Sorting for RNA Sequencing

**Figure 1:**
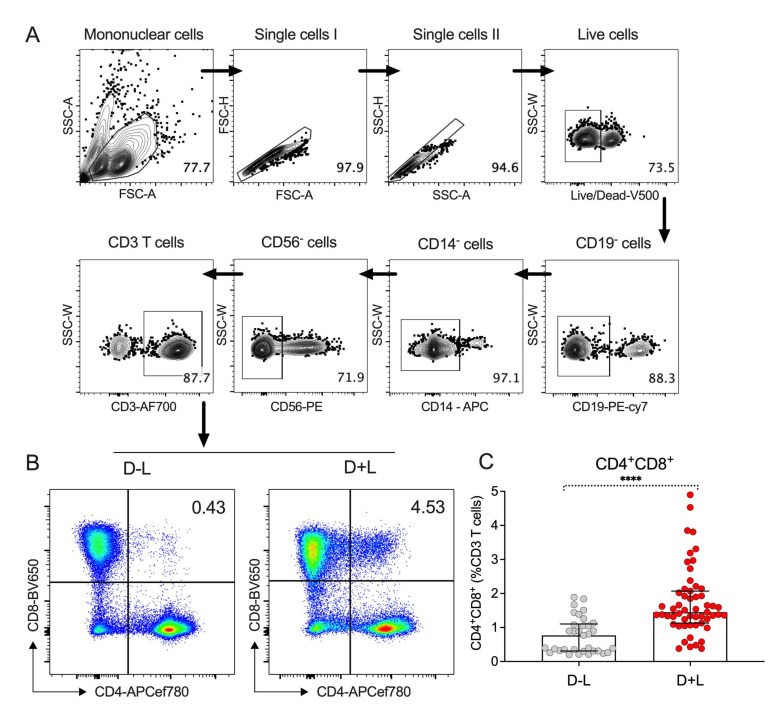
Expansion of CD4^+^CD8^+^ DP population with clinical plasma leakage in dengue disease. (**A**) Gating strategy to identify and sort CD4^+^CD8^+^ DP T cells from human PBMCs isolated from patients who were diagnosed at the acute phase of DENV infection (4–5 days since fever onset). (**B**) Representative flowcytometry plots demonstrate increased percentage of CD4^+^CD8^+^ DP T cells in dengue with plasma leakage (D+L) cohort compared to those in the dengue without plasma leakage (D-L) cohort. (**C**) Bar graph shows the frequencies of CD4^+^CD8^+^ DP T cells in D+L cohort (red, n = 55) and D-L cohort (grey, n = 33). Error bars show median with interquartile range. Statistical analysis was performed by two-tailed Mann–Whitney test, **** *p* < 0.0001.

PBMCs were stained with anti-human CD3, CD4, CD8, CD14, CD19, CD56, and live/dead viability antibodies (see Table S1 for antibody details). Subsequently, live CD4^+^ single positive (SP) (CD14^−^CD19^−^CD56^−^CD3^+^CD4^+^CD8^−^), CD8^+^ SP (CD14^−^CD19^−^CD56^−^CD3^+^CD4^−^CD8^+^), and CD4^+^CD8^+^ DP (CD14^−^CD19^−^CD56^−^CD3^+^CD4^+^CD8^+^) cells were sorted into TRIzol LS Reagent (Invitrogen, Carlsbad, CA, USA) using a BD FACSAria cell sorter (BD Biosciences, San Jose, CA, USA). Flow cytometry data were analyzed by FlowJo X Software (version 10, Tree Star, Ashland, OR, USA). The gating strategy utilized were shown in Figure 1A,B, and results from representative donors from both D-L and D+L groups are shown in Figure 1B. To note that in this study, B cells, monocytes, and NK cells were removed by flowcytometry using CD19, CD14, and CD56 antibodies, and only CD3^+^ T cells were analyzed. However, the roles of these subsets of immune cells may be important as well and warrant further investigation because several relevant factors (e.g., NF-kB) have been associated with plasma leakage [16].

### 2.4. RNA Sequencing

Total RNA was purified and quantified, as previously described [17]. Purified total RNA (5 ng) was amplified following the Smart-seq2 protocol [18]. cDNA was purified using AMPure XP beads (1:1 ratio; Beckman Coulter, Carlsbad, CA, USA). From this step, 1 ng cDNA was used to prepare a standard Nextera XT sequencing library (Nextera XT DNA library preparation kit and index kit (set B and C, respectively), Illumina, San Diego, CA, USA). Samples were sequenced in 3 batches using a NovaSeq 6000 system (Illumina, San Diego, CA, USA) to obtain 50-bp paired-end reads. Both whole transcriptome amplification and sequencing library preparations were performed in a 96-well format to reduce assay-to-assay variability. Quality control steps were included to determine total RNA quality and quantity, the optimal number of PCR pre-amplification cycles, and fragment size selection using the Agilent 2100 Bioanalyzer system (Agilent, Santa Clara, CA, USA). Samples that failed quality control were eliminated from further downstream steps. Barcoded Illumina sequencing libraries (Nextera, Illumina, San Diego, CA, USA) were generated utilizing the automated platform (Biomek FXp, Beckman Coulter, Carlsbad, CA, USA). Libraries were sequenced on the NovaSeq 6000 Illumina platform to obtain 50-bp paired-end reads using the NovaSeq 6000 S4 Reagent kit v1.5 (Illumina, San Diego, CA, USA), generating a median of 25.4 million mapped reads per sample.

### 2.5. Transcriptomic Analysis

Interventionary studies involving animals or humans and other studies that require ethical approval must list the authority that provided approval and the corresponding ethical approval code.

The single-end reads that passed Illumina filters were filtered for reads aligning to tRNA, rRNA, adapter sequences, and spike-in controls. The reads were then aligned to UCSC hg19 reference genome using TopHat (v 1.4.1) [19]. DUST scores were calculated with PRINSEQ Lite (v 0.20.3) [20], and low-complexity reads (DUST > 4) were removed from the BAM files. The alignment results were parsed via the SAMtools [21] to generate SAM files. Read counts to each genomic feature were obtained with the HTseq-count program (v 0.6.0) [22] using the “union” option.

After removing absent features (zero counts in all samples), the raw counts from 3 batches were combined with batch effect removed by BEER v0.1.8 for R [23] and imported to R/Bioconductor package Seurat 4.0 [24] for normalization. Clustering analyses were performed using UMAP for dimensionality reduction. Differentially expressed (DE) genes among samples were identified, with *p*-values calculated by binomial test and adjusted for multiple comparison by the Benjamini–Hochberg algorithm [25] to control the false discovery rate. Significant DE genes were defined as both adjusted *p*-value of < 0.05 and the absolute value of log2 fold-change > 2, and results were presented as volcano plots and heatmaps. Information of DE genes and protein products was extracted from UniProt Consortium [26]. Enrichment analyses were performed on significant DE genes by the R package gprofiler2 [27].

### 2.6. Statistical Analysis

Flow cytometry and clinical data were analyzed by GraphPad Prism Version 8 (La Jolla, CA, USA). The statistical details of the experiments are provided in the respective figure legends. Normality of distribution was assessed by Shapiro–Wilk test. Comparison of the non-parametric continuous data between D+L and D-L cohorts was performed by two-tailed Mann–Whitney test, and categorical data were compared with Fisher’s exact test. Multivariate logistic regression was performed in R (version 4.0.2) (R Development Core Team, Vienna, Austria). Non-parametric data in this study are represented as median with interquartile range. *p*-Values < 0.05 (after adjustment if indicated) were considered statistically significant.

## 3. Results

### 3.1. Expansion of CD4^+^CD8^+^ DP T Cells Is Associated with Risk of Plasma Leakage in Dengue

A total of 88 dengue patients, including 55 with plasma leakage (D+L) and 33 without plasma leakage (D-L) were enrolled in the study and recruited from the North Colombo Teaching Hospital, Ragama in Gampaha District, Sri Lanka and the National Institute of Infectious Diseases, Gothatuwa, Angoda, Sri Lanka between 2010 and 2016. Demographic and clinical information of patient groups included in the analysis are summarized in Table 1 and described in details in methods. It is important to emphasize that the samples studied corresponded to blood draws obtained before any plasma leakage was detectable clinically or radiologically.

Initial phenotyping of peripheral blood mononuclear cells (PBMC) from the two groups (D+L and D-L) was determined by flow cytometry. Specifically, the proportions of CD4^+^CD8^+^ double-positive (DP), CD4^+^ single-positive (SP), and CD8^+^ SP T cells in acute DENV infection was assessed, using the gating strategy illustrated in Figure 1A. Representative donors from both D-L and D+L group are illustrated in Figure 1B. As shown in Figure 1C, we observed a significant higher proportion (*p* < 0.0001) of CD4^+^CD8^+^ DP T cells in the D+L group (median 1.45%, interquartile range (IQR): 1.13–2.07%) compared to the D-L group (median 0.77%, IQR: 0.31–1.10%), with no differences detected for CD4^+^ or CD8^+^ SP T cells (Figure S1). The detailed values of median and IQR for CD4^+^CD8^+^ DP T cells from two cohorts are summarized in Table 1.

To further evaluate the association between the proportion of CD4^+^CD8^+^ DP cells and plasma leakage, we conducted a multivariate logistic regression analysis accounting for other confounding factors, including demographic features (e.g., age, gender), previous DENV infection, as well as hematological and biochemical parameters (e.g., platelet count, hematocrit, AST, and ALT). As shown in Table 2, the percentage of CD4^+^CD8^+^ DP cells was the only significant factor (*p* = 0.002), positively correlated with plasma leakage (log (OR) = 1.86). These results suggested that the CD4^+^CD8^+^ DP T cell population is a novel and relevant marker of development of plasma leakage.

### 3.2. The Gene Expression Profile of CD4^+^CD8^+^ DP T Cells Reveals a Higher Similarity to CD8^+^ T Cells as Opposed to CD4^+^ T Cells

**Figure 2:**
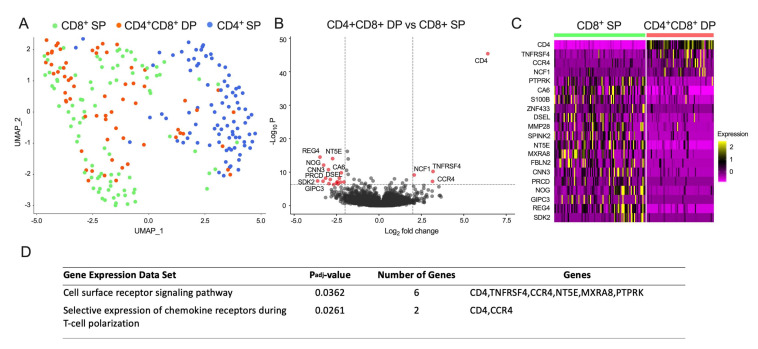
Transcriptomic analysis of CD4^+^CD8^+^ DP T cells compared with CD4^+^ and CD8^+^ SP T cells in acute DENV infection. (**A**) UMAP plot of bulk RNA-seq datasets from acute DENV infection samples colored by cell group (after QC: n = 64 for CD4^+^CD8^+^ DP T cell group, n = 88 for CD4^+^ SP T cell group, and n = 87 for CD8^+^ SP T cell group), where each dot represented one RNA-seq data from one individual. (**B**) Volcano plot shows log_2_ fold change versus −log_10_ *p*-value for the comparison between CD4^+^CD8^+^ DP T cells and CD8^+^ SP T cells. The subset of genes with log_2_ fold change greater than 2 or less than −2 and adjusted *p*-value less than 0.05 are considered significant and indicated by dotted lines. (**C**) Heatmap shows the expression values after variance stabilizing transformation of the significant DE genes found between CD4^+^CD8^+^ DP T cells and CD8^+^ SP T cells. (**D**) Functional pathway enrichment analysis using g:Profiler for differentially expressed genes between CD4^+^CD8^+^ DP and CD8^+^ SP T cells; *p*_adj_ value means adjusted *p* value.

To investigate the nature of CD4^+^CD8^+^ DP cells in acute DENV infection, we further characterized their transcriptomic profiles and compared with CD4^+^ or CD8^+^ single-positive (SP) cells. Cell subsets were sorted and RNA-seq performed, followed by clustering analyses using UMAP for dimensionality reduction. As shown in Figure 2A, at the transcriptomic level, CD4^+^CD8^+^ DP T cells were well resolved from CD4^+^ SP T cells but co-clustered with CD8^+^ SP T cells. This suggests that at the transcriptional level the CD4^+^CD8^+^ DP T cell population is most closely related to CD8^+^ SP T cells.

This result was confirmed by the analysis of which specific genes were differentially expressed (DE) between the various T-cell populations. Overall, there were 20 DE genes (absolute log_2_ fold change (FC) > 2, and adjusted *p*-value (false discovery rate, FDR) < 0.05) identified between CD4^+^CD8^+^ DP and CD8^+^ SP T cells (Figure 2B,C, Table 3) compared to 109 DE genes identified between CD4^+^CD8^+^ DP and CD4^+^ SP T cells (Figure S2 and Table S2). As expected, we observed increased expression of the CD8 (α and β) and CD4 genes by CD4^+^CD8^+^ DP T cells compared to CD4^+^ and CD8^+^ SP T cells, respectively.

### 3.3. Molecular Pathways Associated with CD4^+^CD8^+^ DP T Cells

The analysis above suggested that CD4^+^CD8^+^ DP T cells are more closely related to CD8^+^ SP T cells in terms of their transcription profile. We next set out to examine which genes might be differentially expressed between the two T-cell compartments and might therefore define the specific gene expression pattern associated with CD4^+^CD8^+^ DP T cells. Compared to CD8^+^ SP T cells, we mainly observed in CD4^+^CD8^+^ DP T cells a higher expression of genes involved in T-cell activation (*TNFRSF4*, Tumor necrosis factor receptor superfamily member 4 or OX40), immune cell recruitment (*CCR4*, C-C chemokine receptor type 4), and oxygen dependent phagocytosis (*NCF1*, Neutrophil cytosol factor 1).

Significantly down-regulated DE genes in CD4^+^CD8^+^ DP T cells (Figure 2B,C) included *PTPRK* (Receptor-type tyrosine-protein phosphatase kappa), *CA6* (Carbonic anhydrase 6), *S100B* (S100 Calcium-Binding Protein B), *ZNF433* (Zinc finger protein 433), *DSEL* (Dermatan-sulfate epimerase-like protein), *MMP28* (Matrix metalloproteinase-28), *SPINK2* (Serine protease inhibitor Kazal-type 2), *NT5E* (5′-nucleotidase), *MXRA8* (Matrix remodeling-associated protein 8), *FBLN2* (Fibulin-2), *CNN3* (Calponin-3), *PRCD* (Photoreceptor disk component), *NOG* (Noggin), *GIPC3* (PDZ domain-containing protein), *REG4* (Regenerating islet-derived protein 4), and *SDK2* (Protein sidekick-2), representing intricate processes of cell differentiation and signaling in CD4^+^CD8^+^ cells (Table 3).

The functional pathway enrichment analysis using g:Profiler revealed that those DE genes were enriched in two pathways involved in cell surface receptor signaling pathway (*CD4, TNFRSF4, CCR4, NT5E, MXRA8, PTPRK*) and selective expression of chemokine receptors during T-cell polarization (*CD4, CCR4*) (Figure 2D). These results suggest that the transcriptomic profile of CD4^+^CD8^+^ DP T cells is more closely related CD8^+^ SP T cells with some differences detected.

### 3.4. Paucity of Transcriptomic Changes in CD4^+^CD8^+^ DP T Cells between Patients with and without Plasma Leakage in Dengue

**Figure 3:**
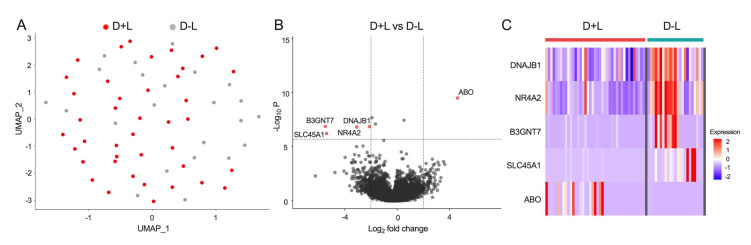
Comparison of transcriptomic signatures of CD4^+^CD8^+^ DP T cells from dengue with plasma leakage (D+L) cohort and dengue without plasma leakage (D-L) cohort. (**A**) UMAP plot of bulk RNA-seq datasets from acute DENV infection samples colored by disease groups (after QC: n = 41 for D+L group, n = 23 for D-L group), where each dot represented one RNA-seq data from one individual. (**B**) Volcano plot shows log_2_ fold change versus −log_10_ *p*-value for the comparison between CD4^+^CD8^+^ DP T cells from D+L and D-L groups. The subset of genes with log_2_ fold change greater than 2 or less than −2 and adjusted *p*-value less than 0.05 are considered significant and indicated by dotted lines. (**C**) Heatmap shows the expression values after variance stabilizing transformation of the significant DE genes found between CD4^+^CD8^+^ DP T cells from D+L and D-L groups.

To further investigate the role of CD4^+^CD8^+^ DP T cells in the pathogenesis of plasma leakage in dengue, we compared transcriptomic profiles between the D+L and D-L groups during the acute febrile phase. CD4^+^CD8^+^ DP T cells isolated from both D+L and D-L groups displayed very similar gene expression profiles and clustered closely to one another based on UMAP analysis (Figure 3A).

Only five significant DE genes were identified (Figure 3B,C), which included in D+L group the down-regulated genes *DNAJB1* (DnaJ homolog subfamily B member 1), *NR4A2* (Nuclear receptor subfamily 4 group A member 2), *B3GNT7* (UDP-GlcNAc:betaGal beta-1,3-N-acetylglucosaminyltransferase), and *SLC45A1* (Proton-associated sugar transporter A) involved in gene expression and post-translational protein modification as well as the up-regulated gene *ABO* (Histo-blood group ABO system transferase) gene (Table 4).

## 4. Discussion

This study provided several novel observations on the host immune response during the acute phase of DENV infection. First, the report that the frequency of a novel CD4^+^CD8^+^ DP T cell subset was increased in D+L versus D-L, and it is thus associated with risk of plasma leakage in dengue disease. Second, on the basis of their transcriptomic profile, CD4^+^CD8^+^ DP T cells were most closely related to CD8^+^ SP T cells, but still, we were able to define 20 DE genes enriched in two functional pathways representative of selective expression of chemokine receptors and signaling on the cell surface during T-cell polarization. Third, while the transcriptomic profile of the CD4^+^CD8^+^ DP T cells was rather similar in D+L and D-L patients, five DE genes were identified.

CD4^+^CD8^+^ DP T cells have been described in several pathological conditions in comparison to normal individuals [28]; for example, expansion of DP cells has been observed in autoimmune diseases (e.g., Myasthenia gravis, Rheumatoid arthritis, multiple sclerosis, Kawasaki diseases, and systemic sclerosis), infectious diseases (e.g., HIV and EBV), and cancers (e.g., melanoma, lymphoma) [28]. However, their phenotype and role in DENV infection have not been reported. Peripheral CD4^+^CD8^+^ DP T cells are a phenotypically and functionally heterogeneous population depending on their origin and pathologic context, and their roles in the pathogenesis of autoimmune diseases, viral infections, and cancers are under ongoing debate [29]. While CD4^+^CD8^+^ DP T cells appear to function at peripheral sites as potent immune suppressors [30,31] or cells with high cytotoxic potential [32,33], a more thorough examination of their contribution to the adaptive immunity in human diseases is warranted [29].

Here, we observed that an increased frequency of CD4^+^CD8^+^ DP T cells during acute DENV infection was associated with development of plasma leakage in dengue disease. Furthermore, CD4^+^CD8^+^ DP T cells appear to be the most significant factor associated with development of plasma leakage even after adjusting for other reported risk factors, such as age, gender, platelet count, hematocrit, AST, ALT levels, and secondary dengue infection [10]. These results indicate that CD4^+^CD8^+^ DP T cells are a novel factor that warrants further investigation, especially taking into consideration of the potential implication in clinical settings and pathophysiology studies of plasma leakage (both onset and severity) in DENV infection by collecting bloods at various time points in future. In particular, the current study did not address the viral load in the blood nor the antigen specificity of the CD4^+^CD8^+^ DP T cells, and future studies will have to address their correlation with the immune response and what fraction of these CD4^+^CD8^+^ DP T cells is actually DENV specific.

In addition, the origin and function of peripheral CD4^+^CD8^+^ DP T cells in various pathological conditions are not fully understood to date. Parrot et al. reported that intra-melanoma DP T cells were transcriptome-wise closer to CD8^+^ SP T cells [34], while Mucida et al. found mature CD4^+^ T helper cells could undergo transcriptomic re-programming and generate cytotoxic CD4^+^CD8^+^ DP T cells after persistent ovalbumin antigen stimulation [35]. However, those studies only investigated limited genes by microarray, and there was no systemic RNA-sequencing (RNA-seq) data comparing transcriptomic profiles of CD4^+^CD8^+^ DP with CD4^+^ or CD8^+^ SP T cells.

Our study reports the first set of RNA-seq data of CD4^+^CD8^+^ DP T cells, and the finding that their transcriptomic profile is most closely related to CD8^+^ SP T cells in acute DENV infection. The principal transcripts distinguishing CD4^+^CD8^+^ DP T cells from CD8^+^ SP T cells encoded OX40 and CCR4, which were found up-regulated in CD4^+^CD8^+^ DP compared to CD8^+^ SP T cells. Interestingly, OX40 has been established as a crucial signaling molecule during persistent viral infection [36] and implicated in their pathology through maintenance of higher numbers of long-lived CD8^+^ effector T cells [37]. In addition, a differential regulation of the expression of the OX40 signaling pathway has been observed in asymptomatic dengue cases compared with clinical cases [38] and associated with dengue disease severity [39]. Moreover, clinical studies in endemic areas have described the associations between the levels of CC chemokines and dengue disease outcome [40,41]. In particular, the CCR4 receptor has been shown to contribute to the pathogenesis of severe conditions [42,43], including dengue [44]. Collectively, our results indicate that CD4^+^CD8^+^ DP T cells are effector T cells that are transcriptome-wise closer to CD8^+^ SP T cells during acute DENV infection.

While the gene expression profile of CD4^+^CD8^+^ DP T in D+L and D-L was relatively similar, DE genes were identified. Interestingly, the ABO gene was the only up-regulated gene, associated with plasma leakage during acute DENV infection. This gene encodes A and B antigens of human blood group and is autosomal dominant [45]. Studies have shown that ABO blood group plays a role in viral infection and host susceptibility, such as norovirus, rotavirus, HIV, influenza viruses, as well as SARS coronavirus (SARS-CoV) [45]. Kalayanarooj et al. found that blood group AB was associated with increased risk of severe dengue disease with plasma leakage in secondary infections [46], consistent with our findings. These differentially expressed genes, together with those discriminating CD4^+^CD8^+^ DP T cells from CD8^+^ SP T cells, could have clinical utility, perhaps as PCR-based markers.

Finally, it can be noted that the results presented herein do not support the previous postulate of original antigenic sin and altered responses [47,48] as key drivers of DENV immunopathology. Rather, they can be interpreted in a growing body of studies [49,50,51,52] that indicate that the T-cell responses and T-cell subsets are modulated in DENV disease and that differential disease severity is mostly associated with differences in magnitude of responses rather than phenotypic differences of different T-cell compartments modulated in the course of infection and disease. That is, severe disease is characterized by an infected cell mass that is larger than that of mild disease, and an enhanced antigen mass stimulates a greater magnitude of T-cell responses.

## 5. Conclusions

Plasma leakage in dengue diseases is a warning complication which requires early detection and clinical intervention. In this study, we observed increased frequency of a novel CD4^+^CD8^+^ double-positive (DP) T-cell subset in the acute febrile phase of the DENV infection is associated with risk of developing plasma leakage later in dengue disease. Further transcriptomic analysis suggested this T-cell subset is closest to CD8^+^ single-positive (SP) T cells. Nonetheless, the transcriptomic profile of the CD4^+^CD8^+^ DP T cells was largely similar in patients with and without plasma leakage, implying a role of CD4^+^CD8^+^ DP cells in plasma leakage through a quantitative increase rather than functional alteration. These data provide new insight into the host immune response in DENV infection and the role of CD4^+^CD8^+^ DP T cells in the pathogenesis of plasma leakage.

## Figures and Tables

**Table 1:** Demographic and clinical information of Sri Lanka dengue patients.

Cohorts	D-L	D+L	*p* value	Total
No. of subjects	33	55	-	88
Age (years)	26.0 (19.0–43.0)	29.0 (23.7–35.5)	0.465	29.0 (22.0–37.0)
Gender (% female)	30.3%	42.6%	0.267	37.9%
Days of acute blood sample collection from fever onset	5.0 (4.0–6.0)	4.0 (4.0–6.0)	0.246	4.0 (4.0–6.0)
Dengue tests (% positive)				
IgM	57.6%	66.7%	0.371	63.2%
IgG	72.7%	76.4%	0.800	75.0%
PCR	30.3%	49.0%	0.118	41.7%
Secondary dengue infection (%)	72.7%	76.4%	0.800	75.0%
Platelet count (×1000/mm^3^)	74.0 (36.0–120.0)	39.0 (13.0–90.0)	0.007 **	59.0 (20.7–102.8)
Hematocrit (%)	40.9 (37.2–44.5)	42.3 (39.5–46.1)	0.139	42.1 (37.4–44.8)
AST (U/L)	113.0 (43.5–216.7)	230.0 (96.0–309.7)	0.017 *	176.0 (66.5–300.1)
ALT (U/L)	90.5 (32.2–168.6)	150.0 (62.0–280.4)	0.028 *	111.9 (50.0–226.5)
%CD4^+^CD8^+^ DP	0.77 (0.31–1.10)	1.45 (1.13–2.07)	<0.0001 ****	1.31 (0.61–1.67)

D-L, dengue without plasma leakage; D+L, dengue with plasma leakage; PCR, polymerase chain reaction; AST, aspartate aminotransaminase; ALT, alanine aminotransferase; DP, double positive, -, not calculated; continuous data are expressed as median and interquartile range, *p*-values were compared between D-L and D+L groups, * *p* < 0.05, ** *p* < 0.01, **** *p* < 0.0001.

**Table 2:** Multivariate analysis of risk factors associated with plasma leakage in dengue disease by a logistic regression model.

Risk Factors	Estimate (β)	Standard Error	Z Score	*p*_adj_ Value
%CD4^+^CD8^+^ DP	1.8632	0.5946	3.133	0.002 **
Age (years)	0.0151	0.0222	0.682	0.495
Gender (M/F)	−0.7616	0.8402	−0.907	0.365
Secondary dengue (Y/N)	−0.1705	0.8201	−0.208	0.835
Platelet count (×1000/mm^3^)	−0.0105	0.0082	−1.280	0.200
Hematocrit (%)	0.0510	0.0766	0.666	0.505
AST (U/L)	0.0011	0.0031	0.369	0.712
ALT (U/L)	0.0001	0.0029	0.042	0.966

DP, double positive; M/F, male/female; Y/N, yes/no; AST, aspartate aminotransaminase; ALT, alanine aminotransferase, *p*_adj_ value, adjusted *p* value; Akaike information criterion (AIC): 81.106, ** *p* < 0.01.

**Table 3:** List of DE genes found between CD4^+^CD8^+^ DP and CD8^+^ SP cells.

Gene Symbol	Encoded Protein	*p*-Value	Log_2_FC	FDR	Gene Product Function *
Higher expression in CD4^+^CD8^+^ DP cells					
CD4	T-cell surface glycoprotein CD4	4.11E-46	6.42	2.40E-41	Cell differentiation antigen CD4, MHC class II receptor
TNFRSF4	Tumor necrosis factor receptor superfamily member 4 (OX40)	6.24E-11	3.19	3.64E-06	Costimulatory molecule implicated in long-term T-cell immunity
CCR4	C-C chemokine receptor type 4 (CCR4)	5.80E-08	3.16	3.38E-03	High-affinity binding for basophil chemoattractant, G protein coupled receptor superfamily, specific receptor for thymus and activation-regulated chemokine
NCF1	Neutrophil cytosol factor 1	7.09E-10	2.08	4.14E-05	Activate NADPH oxidase for superoxide production and oxygen dependent mechanism of phagocytosis
**Reduced expression in CD4^+^CD8^+^ DP cells**					
PTPRK	Receptor-type tyrosine-protein phosphatase kappa	9.03E-08	−2.06	5.27E-03	Transmembrane receptor protein tyrosine phosphatase activity, regulation of processes involving cell contact and adhesion
CA6	Carbonic anhydrase 6	1.58E-10	−2.22	9.20E-06	Carbonate dehydratase activity, protein binding
S100B	S100 Calcium-Binding Protein B	6.52E-09	−2.33	3.81E-04	Calcium ion binding, protein homodimerization activity
ZNF433	Zinc finger protein 433	3.47E-07	−2.42	2.03E-02	Transcriptional regulation, RNA polymerase II regulatory region sequence-specific DNA binding
DSEL	Dermatan-sulfate epimerase-like protein	7.12E-08	−2.45	4.15E-03	Membrane proteins, sulfotransferase activity, isomerase activity
MMP28	Matrix metalloproteinase-28	1.62E-07	−2.50	9.48E-03	Tissues homeostasis and repair, metalloendopeptidase activity
SPINK2	Serine protease inhibitor Kazal-type 2	5.22E-07	−2.67	3.04E-02	Serine-type endopeptidase inhibitor activity
NT5E	5′-nucleotidase	9.12E-15	−2.73	5.32E-10	Hydrolyzes extracellular nucleotides into membrane permeable nucleosides
MXRA8	Matrix remodeling-associated protein 8	1.56E-08	−2.87	9.13E-04	Transmembrane protein which can modulate activity of various signaling pathways
FBLN2	Fibulin-2	2.67E-07	−2.95	1.56E-02	Extracellular matrix protein, expressed in elastic tissues including intima of blood vessels
CNN3	Calponin-3	1.82E-11	−2.98	1.06E-06	Cytoskeletal protein involved in cell-cell adhesion, downregulated during dedifferentiation of vascular smooth muscle cell
PRCD	Photoreceptor disk component	6.21E-09	−3.15	3.62E-04	Opsin binding
NOG	Noggin	7.99E-13	−3.26	4.66E-08	Cytokine binding, protein homodimerization activity
GIPC3	PDZ domain-containing protein	5.15E-08	−3.30	3.00E-03	Protein binding
REG4	Regenerating islet-derived protein 4	3.15E-15	−3.48	1.84E-10	Carbohydrate binding, signaling receptor activity
SDK2	Protein sidekick-2	4.31E-08	−3.62	2.52E-03	Adhesion molecule

Log_2_FC, log_2_ fold change; FDR, false-discovery rate; DE genes cut off: FDR < 0.05 and Log_2_FC > 2, * Gene protein products with functional annotation information was extracted from UniProt Consortium [26].

**Table 4:** List of DE genes found between CD4^+^CD8^+^ DP cells of D+L and D-L cohorts.

Gene Symbol	Encoded Protein	*p*-Value	Log_2_FC	FDR	Gene Product Function *
Reduced expression in CD4^+^CD8^+^ DP cells of D+L cohort					
DNAJB1	DnaJ homolog subfamily B member 1	1.36E-07	−2.12	7.96E-03	ATPase activator activity, transcription corepressor activity, Hsp70 protein binding, involved in protein folding
NR4A2	Nuclear receptor subfamily 4 group A member 2	1.49E-07	−3.08	8.72E-03	RNA polymerase II regulatory region sequence-specific DNA binding
B3GNT7	UDP-GlcNAc:betaGal beta-1,3-N-acetylglucosaminyltransferase	1.31E-07	−5.48	7.66E-03	Acetylglucosaminyltransferase activity, protein binding
SLC45A1	Proton-associated sugar transporter A	6.06E-07	−5.39	3.53E-02	Sucrose:proton symporter activity
**Higher expression in CD4^+^CD8^+^ DP cells of D+L cohort**					
ABO	Histo-blood group ABO system transferase	3.11E-10	4.62	1.82E-05	Glycosyltransferase activity

Log_2_FC, log_2_ fold change; FDR, false-discovery rate; DE genes cut off: FDR < 0.05 and Log_2_FC > 2, * Gene protein products with functional annotation information was extracted from UniProt Consortium [26].

## Data Availability

The RNA-Seq data were deposited in the NCBI’s Gene Expression Omnibus (GEO) database under the accession code GSE178240. The other datasets generated and/or analyzed during the current study are available from the corresponding author on reasonable request.

## Supplementary Materials

The following are available online at https://www.mdpi.com/article/10.3390/v14010090/s1, Figure S1: Distribution of CD4+ and CD8+ single positive (SP) in D-L and D+L cohorts; Figure S2: Transcriptomic analysis of CD4^+^CD8^+^ DP T cells compared with CD4^+^ SP T cells in acute DENV infection; Table S1: List of antibodies used in the flowcytometry study; Table S2: List of DE genes found between CD4+CD8+ DP and CD4+ SP cells.
