# Supplementary material for: A Population of CD4+CD8+ Double-Positive T Cells Associated with Risk of Plasma Leakage in Dengue Viral Infection
Source: Viruses. 2022 Jan 5;14(1):90. doi: 10.3390/v14010090 (PMC8779337; doi:10.3390/v14010090)
Supplement: Supplementary file 1 [file viruses-14-00090-s001.zip › Suplemental Figure S1-2 and Table S1.pdf]

**Supplemental Materials for: A population of CD4+CD8+ double-positive T cells associated with risk of plasma leakage in dengue viral infection (Yu *et al.*)**

**Figure S1**

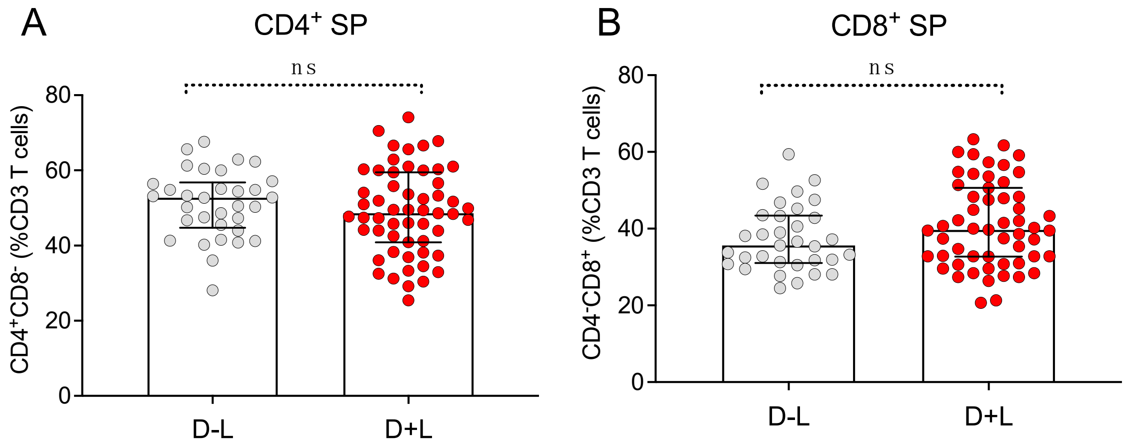


**Figure S1.** Distribution of CD4+ and CD8+ single positive (SP) in D-L and D+L cohorts. (**A**) Bar graph shows the frequencies of $CD4^+$ SP T cells in D+L cohort (n = 55) and D-L cohort (n = 33). (**B**) Bar graph shows the frequencies of $CD8^+$ SP T cells in D+L cohort (n = 55) and D-L cohort (n = 33). Error bars show median with interquartile range. Statistical analysis was performed by two-tailed Mann-Whitney test, ns means statistically non-significant (P > 0.05).

# Figure S2

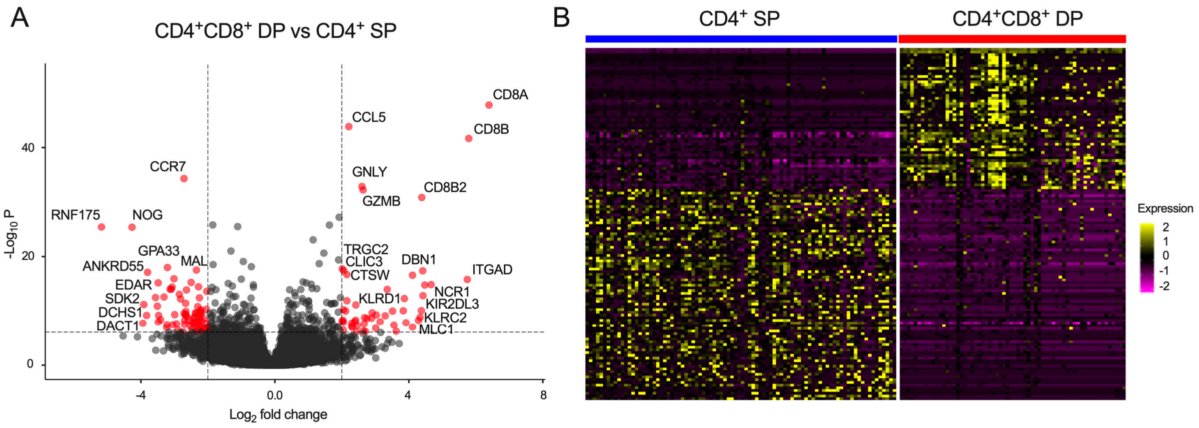


**Figure S2.** Transcriptomic analysis of $CD4^{+}CD8^{+}$ DP T cells compared with $CD4^{+}$ SP T cells in acute DENV infection. (**A**) Volcano plot shows $\log_2$ fold change versus $-\log_{10}$ P value for the comparison between $CD4^{+}CD8^{+}$ DP T cells (n = 64) and $CD4^{+}$ SP T cells (n = 88). The subset of genes with $\log_2$ fold change greater than 2 or less than -2 and adjusted P value less than 0.05 are considered significant and indicated by dotted lines. (**B**) Heatmap shows the expression values after variance stabilizing transformation of the significant DE genes found between $CD4^{+}CD8^{+}$ DP T cells and $CD4^{+}$ SP T cells.

**Table S1.** List of antibodies used in the flowcytometry study.

| Antibody | Fluorochrome | Clone | Vendor | Catalog number |
|---|---|---|---|---|
| CD3 | AF700 | UCHT1 | Invitrogen | 56-0038-42 |
| CD4 | APCef780 | RPA-T4 | Invitrogen | 47-0049-42 |
| CD8 | BV650 | RPA-T8 | Biolegend | 301042 |
| CD14 | APC | 61D3 | TonBo Biosciences | 20-0149-T100 |
| CD19 | PE-Cy7 | HIB19 | TonBo Biosciences | 60-0199-T100 |
| CD56 | PE | CMSSB | Life-Tech | 12-0567-42 |
| Live/Dead Viability | eF506/Aqua | - | Invitrogen | 65-0866-18 |

**Table S2.** List of DE genes found between $CD4^+CD8^+$ DP and $CD4^+$ SP cells.

Attached as a separate excel file: Supplemental Table S2.xlsx
